# *Baccharis* Species Essential Oils: Repellency and Toxicity against Yellow Fever Mosquitoes and Imported Fire Ants

**DOI:** 10.3390/jox13040041

**Published:** 2023-10-27

**Authors:** Abbas Ali, Farhan Mahmood Shah, Jane Manfron, Luciane M. Monteiro, Valter P. de Almeida, Vijayasankar Raman, Ikhlas A. Khan

**Affiliations:** 1 National Center for Natural Products Research, The University of Mississippi, University, MS 38677, USA; fshah@olemiss.edu (F.M.S.); ikhan@olemiss.edu (I.A.K.); 2 Postgraduate Program in Pharmaceutical Sciences, State University of Ponta Grossa, Ponta Grossa 84030-900, Brazil; janemanfron@hotmail.com (J.M.); lucianemendesmonteiro@gmail.com (L.M.M.); valterpaesdealmeida@gmail.com (V.P.d.A.)

**Keywords:** *Baccharis*, kongol, spathulenol, *Solenopsis invicta*, mosquitoes, essential oil

## Abstract

Essential oils from five *Baccharis* species were screened for their toxicity and biting deterrence/repellency against yellow fever mosquito, *Aedes aegypti* (L.), and imported fire ants, including *Solenopsis invicta* Buren (RIFA), *Solenopsis richteri* Forel (BIFA) and their hybrids (HIFA). *Baccharis microdonta* DC. and *B. punctulata* DC. at 10 µg/cm^2^ showed biting deterrence similar to DEET, *N*, *N*-diethyl-*meta*-toluamide at 25 nmol/cm^2^, whereas the repellency of *B. pauciflosculosa* DC., *B. sphenophylla* Dusén ex Malme and *B. reticularioides* Deble & A.S. Oliveira essential oils was significantly lower than DEET against mosquitoes. Two major compounds from the active essential oils, kongol and spathulenol, also exhibited biting deterrence similar to DEET against mosquitoes. The highest toxicity exhibited against mosquitoes was by *Baccharis punctulata* essential oil (LC_50_ = 20.4 ppm), followed by *B. pauciflosculosa* (LC_50_ = 31.9 ppm), *B. sphenophylla* (LC_50_ = 30.8 ppm), *B. microdonta* (LC_50_ = 28.6 ppm), kongol (LC_50_ = 32.3 ppm), spathulenol (LC_50_ = 48.7 ppm) and *B. reticularioides* essential oil (LC_50_ = 84.4 ppm). *Baccharis microdonta* essential oil showed repellency against RIFA, BIFA and HIFA at 4.9, 4.9 and 39 µg/g, respectively. *Baccharis microdonta* essential oil also showed toxicity with LC_50_ of 78.9, 97.5 and 136.5 µg/g against RIFA, BIFA and HIFA, respectively, at 24 h post treatment.

## 1. Introduction

Mosquitoes are vectors of many human and animal diseases [1]. These diseases result in high human mortality, especially in underdeveloped countries. The use of synthetic chemicals to manage mosquito populations is common and documented to be an effective approach for reducing mosquito-borne disease incidence [2]. Their repeated, long-term use, however, has led to the development of resistance in mosquitoes [3]. Insect repellents prevent mosquitoes from biting humans, resulting in the reduction of disease incidence [4]. Repellents are commonly used against mosquitoes to minimize the incidence of diseases by giving localized personal protection. DEET (*N*,*N*-diethyl-*meta*-toluamide) has been present in the market for more than half a century and is a gold standard for measuring the effectiveness of new repellents [5]. Imported fire ants, including red imported fire ant (RIFA) *Solenopsis invicta* Buren, black imported fire ants (BIFA) *S. richteri* Forel (Hymenoptera: Formicidae) and hybrids (HIFA) of these two species are significant pests throughout the southeastern United States, parts of western states, Mexico and Puerto Rico [6]. With increasing trade and transportation, RIFA is now reported from more than 20 countries and territories [7] and is considered the most serious invader [8,9]. Baits and contact synthetic insecticides are primarily used to manage imported fire ants. Repeated use of such interventions can induce behavioral and physiological modifications in the fire ants in the long run [10,11,12] which can make fire ant management even more challenging [13,14].

Because of the risk of insecticide resistance and the potential of environmental contamination by chemicals, our research program is focused on exploring alternative resources to find new insecticides and repellents that can effectively manage these pests. Phytochemicals including plant extracts and essential oils may offer novel alternatives to effectively manage mosquitoes and imported fire ant populations [15,16,17]. Plant-derived natural products are gaining increasing attention as alternatives to synthetic agents in pest management [18,19]. Natural products are renewable sources with low mammalian toxicity, are biodegradable, and are safer to use compared to synthetic counterparts [20].

Many natural products are specialized plant metabolites that show anti-insect activities [21]. The bioactive compounds in natural products may act as attractants, antifeedants, oviposition modifiers or affect key metabolic processes, resulting in rapid death. The discovery of novel insecticides and repellents against pests of great medical importance from relatively non-toxic and biodegradable natural products has been the focus [22]. Many repellents and toxicants sourced from plants [23] are effective against imported fire ants [17,24,25,26].

The genus Baccharis (Asteraceae) comprises about 440 species [27] of subshrubs, shrubs and trees and show a wide range of morphological diversity [27]. Many of the species are commonly used in traditional medicine due to the presence of essential oils with medicinal properties [28,29,30]. Several important biological activities attributed to these essential oils include anti-inflammatory [31], antibacterial, antifungal, antiprotozoal, antiviral [32,33], schistosomicidal [34], antimalarial, antitrypanosomal and insecticidal [35]. Anti-insect activities have been reported for many *Baccharis* species against pests of medical importance. Many bioactive compounds exhibiting toxicity and repellency have been identified. *Baccharis darwinii* essential oil with limonene and 4-terpineol as major compounds were promising repellents against *Triatoma infestans* [36]. *Baccharis reticularia* DC. showed larvicidal activity against *Aedes aegypti* L. [37]. Alves et al. [38] reported that *B. dracunculifolia* essential oil and its major compound (*E*)-nerolidol showed promise as toxicants against *Culex quinquefasciatus* (Culicidae). Lima et al. [39] showed that *B. reticularia* essential oil and its monoterpene constituents (limonene, *α*-pinene and *β*-pinene) based nanoemulsions were effective repellents against *Tribolium castaneum* (Herbst). To the best of our knowledge, no prior report exists on the insecticidal and repellent activities of these *Baccharis* species to imported fire ants.

Given that *Baccharis* is a highly diversified genus, many species have been explored and many are yet to be explored. Recently, we published the chemical composition of five *Baccharis* species essential oils and their insecticidal activities against bed bugs [35]. To the best of our information, there is no reported information regarding the activity of the essential oils of these species of *Baccharis* against mosquitoes and imported fire ants. The present study was aimed at determining the biting deterrence/repellency and toxicity of essential oils of *B. microdonta* DC., *B. pauciflosculosa* DC., *B. punctulate* DC., *B. reticularioides* Deble & A.S. Oliveira and *B. sphenophylla* Dusén ex Malme and their selected pure compounds, against yellow fever mosquito and imported fire ants.

## 2. Results

The major compounds of *B. microdonta* were kongol (22.22%) and spathulenol (22.74%). Limonene (18.77%) and *β*-pinene (18.33%) were the main components of *B. pauciflosculosa* (Table S1). The major component in *B. punctulata* was *α*-bisabolol (23.63%) while *B. reticularioides* contained *α*-pinene (24.50%) as the main component. In *B. sphenophylla*, *α*-pinene (10.74%), spathulenol (13.15%), limonene (14.33%) and *β*-pinene (15.24%) were the major components. Kongol was present only in *B. microdonta* and *α*-bisabolol, the major compound of *B. punctulata*, can be considered as the chemical markers for these species.

### 2.1. Mosquito Bioassays

**Figure 1:**
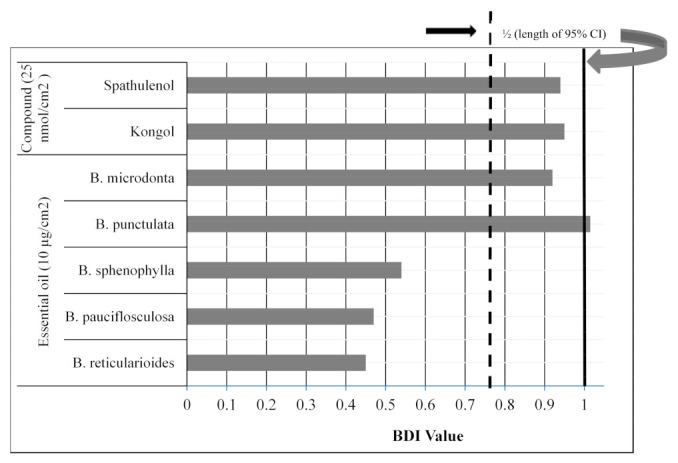
Mean Biting Deterrence Index (BDI) values of the essential oils from *Baccharis* species and their pure compounds against female *Aedes aegypti*. Ethanol was the solvent control and DEET at 25 nmol/cm^2^ was used as the positive control. Mean BDI values falling between 1/2 length of 95% CI and 1 are statistically similar to DEET.

In high throughput K & D bioassay used in these studies, the mosquito’s biting activity depends on the biting deterrent properties of the test material. Mean BDI values of essential oils from five *Baccharis* species and two major compounds against *Ae. aegypti* are given in Figure 1. The biting deterrent activity of *B. microdonta* and *B. punctulata* essential oils at 10 µg/cm^2^ was statistically similar to DEET at 25 nmol/cm^2^, whereas the activity of *B. pauciflosculosa*, *B. sphenophylla* and *B. reticularioides* essential oils was lower than DEET. Biting deterrence of kongol and spathulenol was also similar to DEET.

Toxicity data on the essential oils from *Baccharis* species against *Ae. aegypti* are given in Table 1. *Baccharis punctulata* essential oil with LC_50_ of 20.4 ppm showed the highest toxicity among the materials tested. Toxicity of *B. pauciflosculosa* (LC_50_ = 31.9 ppm), *B. sphenophylla* (LC_50_ = 30.8 ppm), *B. microdonta* (LC_50_ = 28.6 ppm) and kongol (LC_50_ = 31.9 ppm) was similar. Spathulenol showed LC_50_ of 48.7 ppm whereas the lowest toxicity (LC_50_ = 84.4 ppm) was found in *B. reticularioides* essential oil.

### 2.2. Imported Fire Ant Bioassays

For digging bioassays, the mean weight (g) of sand removed by the RIFA, BIFA and HIFA workers is presented in Table 2. Based on the amount of sand removed, *B. microdonta* essential oil showed significantly higher repellency than ethanol at dosages of 19.5–4.9 µg/g against RIFA and BIFA, whereas repellency in HIFA at 19.5 µg/g was similar to ethanol. Repellency of DEET at 156 and 78 µg/g was significantly higher whereas at 39 µg/g, the activity was similar to ethanol.

*Baccharis microdonta* essential oil which showed larvicidal activity against yellow fever mosquitoes was further screened for its toxicity against RIFA, BIFA and HIFA workers. Toxicity data of *B. microdonta* essential oil are shown in Table 3. *Baccharis microdonta* essential oil showed LC_50_ of 78.9, 97.5 and 136.5 µg/g against RIFA, BIFA and HIFA workers, respectively, whereas bifenthrin with LC_50_ of 0.03, 0.32 and 0.018 µg/g, respectively, was more toxic at lower doses at 24 h post treatment. Based on LC_50_ values, the toxicity of *B. microdonta* essential oil was significantly higher in RIFA than in HIFA, whereas no difference was found between BIFA and HIFA or RIFA.

## 3. Discussion

In our ongoing screening program, we tested essential oils and selected major compounds from five species of *Baccharis* against mosquitoes and imported fire ants. Tested species of *Baccharis* drastically varied in chemical composition. In the two active essential oils, kongol was present only in *B. microdonta* and *α*-bisabolol was the major compound of *B. punctulate*. The chemical composition of essential oils is reported to vary throughout the life of the plants. The development of species and the production of chemical compounds are reported to be influenced by environmental factors, circadian rhythms and seasonal growing conditions [35]. Natural products like essential oils and their phytochemical constituents are multitudes of novel chemistries and are reported to have diverse biological activities against insects [40,41]. The mode of action of natural products includes interference with the neuromodulator octopamine [42], GABA receptors [43] or alteration in the activities of AchE [37,38], cytochrome P450s, glutathione S-transferase [44,45] and respiration [46]. Natural products are reported to have repellent and insecticidal activities [47,48], alter the insect growth or suppress the feeding/molting processes [49,50].

In the high throughput bioassay, the mosquito’s biting depends on the biting deterrent properties of the tested materials. Biting deterrent activity of essential oils of *B. microdonta* and *B. punctulata* was similar to DEET whereas the activity in other essential oils was lower. Kongol was present only in the essential oil of *B. microdonta* while spathulenol was one of the major compounds present in the active essential oil [35]. Therefore, these two compounds were also tested. The biting deterrent activity of these two compounds was also similar to DEET. Cantrell et al. [51] reported that the biting deterrent activity of spathulenol was similar to DEET against mosquitoes. The pure compound *α*-bisabolol was not tested in this study because it has been reported to be an active repellent against mosquitoes and imported fire ants [52,53]. This is the first report of the biting deterrent activity of kongol against mosquitoes. The presence of spathulenol and kongol in the essential oil of *B. microdonta* appears to be the cause of the biting deterrence. Spathulenol is one of the major contents of the essential oil of *B. punctulata* and appears to be responsible for the repellency of this essential oil against mosquitoes. Our data corroborates the findings of Gillij et al. [54] who reported the repellency and residual activity of *B. spartioides* (Hook. & Arn. Ex DC.) J. Rémy in Gay, a different species of *Baccharis* against *Ae. aegypti* at a concentration of 50 mg/L.

Many researchers have reported the toxicity of *Baccharis* species against mosquitoes and other insects. Chantraine et al. [55] reported 90% mortality of *Baccharis* sp. Aerial parts essential oil at 50 mg/L against *Ae. aegypti* with LC_50_ of 14.7 mg/L. Alves et al. [38] reported that the *B. dracunculifolia* DC. Essential oil has larvicidal activity (LC_50_ = 34.45 mg/L) against *Culex quinquefasciatus*. In topical application bioassays, essential oil from *B. darwinii* Hook. & Arn. Showed toxicity against *Ceratitis capitata* adults with LC_50_ of 45.2 µg/fly [36]. de Souza et al. [56] reported toxicity of essential oils and the pure compounds from leaves of seven species of *Baccharis* (*B. anomala* DC., *B. calvescens* DC., *B. mesoneura* DC., *B. milleflora* (Less.) DC., *B. oblongifolia* (Ruiz & Pav.) Pers., *B. trimera* (Less.) DC. And *B. uncinella* DC.) against the larvae and adults of *Drosophila suzukii*. Botas et al. [37] reported that the *B. reticularia* DC. Essential oil and major compound limonene-based nanoemulsions, with LC_50_ values of 118.94 μg/mL and 81.19 μg/mL, respectively, were active larvicides against early fourth instar larvae of *A. aegypti*. In our results, *B. punctulata* essential oil (LC_50_ of 20.4 ppm) showed the highest toxicity against one-day-old first instar larvae of *Ae. aegypti*, whereas toxicity of *B. pauciflosculosa* (LC_50_ = 31.9 ppm), *B. sphenophylla* (LC_50_ = 30.8 ppm), *B. microdonta (*LC_50_ = 28.6 ppm) and kongol (LC_50_ = 31.9 ppm) was similar. The lowest toxicity was for *B. reticularioides* essential oil (LC_50_ = 84.4 ppm), followed by spathulenol (LC_50_ = 48.7 ppm). Our study is the first reporting of the toxicity of these essential oils and selected pure compounds against one day old first instar larvae of *Ae. aegypti*.

Fire ants’ digging behavior has been used to measure the repellency in fire ants [17,53,57,58]. In digging bioassay, repellency was determined by comparing the quantity of sand removed in treated sand versus control [53,58]. Based on the ability of a repellent to suppress the digging activity, fire ants can be prevented from invading sensitive areas [59] and as a quarantine treatment to repel fire ants from the nursery stocks and equipment [60,61,62]. Many natural products have been reported to show repellency against imported fire ants. Appel et al. [59] reported repellency of mint oil granules against imported fire ants. Compounds from clove, *Syzygium aromaticum*, cineole and d-camphor from *Artemisia annua* L., and camphor essential oil from *Cinnamonum camphora* Siebold were reported to be significant repellents against RIFA [63,64,65]. Hashimoto et al. [66] showed microencapsulated allyl isothiocyanate to be repellent against RIFA. He et al. [60] showed methyl isoeugenol to repel foraging RIFA significantly longer than eugenol and it repelled nesting ants from nesting inside flowerpots containing treated sand for over a month. To be sure, 1-decanol, 1-octanol and essential oils from *Magnolia grandiflora* showed promise as repellents against HIFA [17]. Our results demonstrated that *B. microdonta* essential oil showed higher repellency than DEET against RIFA, BIFA and HIFA workers, and repellency of this essential oil varied for individual group of imported fire ants. *Baccharis microdonta* essential oil showed significantly higher repellency than ethanol at dosages of 19.5–4.9 µg/g against RIFA and BIFA, whereas the activity at 19.5 µg/g in HIFA was similar to ethanol, suggesting that this essential oil was more active against RIFA and BIFA than HIFA, indicating great potential for its application in imported fire ant management. These findings corroborate with Chen et al. [57], who reported the repellency of callicarpenal and intermedeol, isolated from *Callicarpa americana* L. and *Callicarpa japonica* Thunb. varied with respect to ant species used, callicarpenal and intermedeol showed repellency against RIFA at concentrations as low as 50 ppm and 1.50 ppm, respectively, whereas against BIFA and HIFA showed repellency at 6.25 ppm. Essential oil from *B. microdonta* showed toxicity against all three imported fire ants tested. There were no differences in the repellency of *B. microdonta* essential oil and DEET among the species, whereas toxicity was higher in RIFA followed by BIFA and HIFA. Differences in toxicity among RIFA, BIFA and HIFA demonstrated that the toxicity of various natural products can vary among imported fire ant species/hybrid. There is no prior documented report on the toxicity and repellency of these *Baccharis* species essential oils against imported fire ants. This study is the first detailed report on the repellency and toxicity of *B. microdonta* essential oil against imported fire ants.

## 4. Conclusions

Biting deterrence of essential oils of *B. microdonta* and *B. punctulata* and their two pure compounds was similar to DEET against mosquitoes. Similarly, *B. microdonta* essential oil, spathulenol and kongol were toxic to mosquito larvae. The essential oil of *B. microdonta* showed both repellency and toxicity against imported fire ant workers. Based on these data, these essential oils can be used as toxicants and deterrents/repellents against mosquitoes and imported fire ants. Further studies should explore the repellent/toxicant potential of these essential oils and pure compounds by testing in different formulations against mosquitoes and imported fire ants under elaborated laboratory and field conditions.

## 5. Materials and Methods

### 5.1. Plant Material

Fresh leaves and stems of *Baccharis microdonta*, *B. pauciflosculosa*, *B. punctulata*, *B. reticularioides* and *B. sphenophylla* were collected, in triplicate, from Campos Gerais, Ponta Grossa, Paraná, Southern Brazil (coordinates 25°5′11″ S and 50°6′23″ W) during March 2016. Collection methods followed and details of the voucher specimens are given by Budel et al. [35].

#### 5.1.1. Extraction of Essential Oils

The collected samples were shade dried at room temperature and cut into small pieces (~1 cm). The dried samples were then distilled in a Clevenger-type apparatus for 4 h. The oil obtained from 100 g of dried leaves was separated and dried over anhydrous Na_2_SO_4_. Details of the procedures are described by Budel et al. [35].

#### 5.1.2. Isolation of Compounds and Gas Chromatography-Mass Spectrometry (GC/MS) Analysis

This study is the continuation of a project that was initiated in our ongoing natural products screening program at the National Center for Natural Products Research, University of Mississippi. Chemical compositions of the samples of essential oils used in these studies, GC/MS analysis of leaves and stems of *Baccharis* species, and methods employed for isolation of these compounds are described by Budel et al. [35].

### 5.2. Bioassays

#### 5.2.1. Mosquitoes

*Aedes aegypti* were obtained from the Mosquito and Fly Research Unit at the Center for Medical, Agricultural and Veterinary Entomology, USDA-ARS, Gainesville, FL, USA. Eggs were hatched and the larvae were reared to the adult stage in our laboratory at 27 ± 2 °C and 60 ± 10% RH with a photoperiod regimen of 12:12 h (L:D).

#### 5.2.2. K & D Bioassay

**Figure 2:**
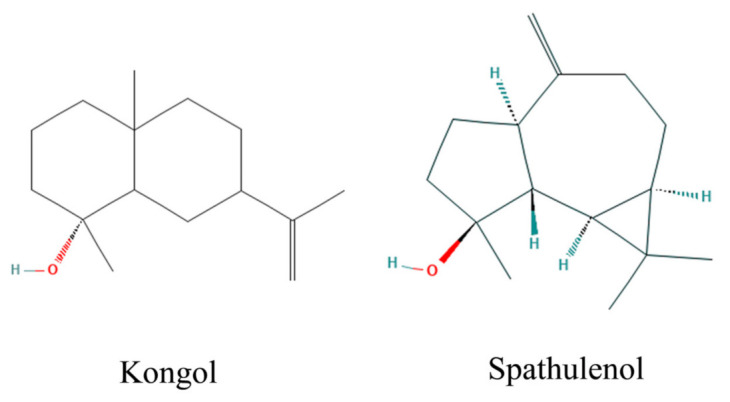
Chemical structures of Kongol and spathulenol.

Bioassays were conducted to determine the biting deterrence of *Baccharis* species essential oils against *Ae. aegypti* by using a K & D bioassay, described by Klun et al. [67]. The bioassay consisted of a module having 6 (3 × 4 cm) wells with a capacity to contain 6 mL of the feeding solution [46]. Green, fluorescent tracer dye (www.blacklightworld.com), accessed on 21 September 2021, was added to the feeding solution to confirm the feeding by the female mosquitoes. Samples of five *Baccharis* species essential oils and pure compounds spathulenol and kongol (Figure 2) were tested for their biting deterrence. *Baccharis* species essential oils were tested at a dose of 10 µg/cm^2^ whereas the pure compounds and DEET were tested at a dose of 25 nmol/cm^2^. The solvent used was a molecular biology grade 100% ethanol (Fisher Scientific, Fair Lawn, NJ, USA). Warm water was constantly circulated through the reservoir to keep a constant temperature of the feeding solution at 37 °C using a circulatory bath. Treatments were randomly allocated to six 4 × 5 cm marked areas of the organdy, which was positioned over the collagen-covered feeding solution. A Teflon separator was used between the treated organdy and the module. K and D module containing five females per cell was placed over treated organdy, and the females were exposed by opening the trap doors. After 3 min of exposure, the number of feeding females biting through treated organdy was recorded after 3 min. To confirm the feeding, mosquitoes were prodded back into the cells and squashed in tissue paper. Each bioassay consisted of 4 treatments, DEET as a positive and ethanol as solvent control. Two sets of 5 replications with 5 females per cell were run on 2 different days using a new batch of females in each replication.

#### 5.2.3. Larval Bioassays

Larvicidal activity of essential oils of *Baccharis* species and its major constituents against *Ae. Aegypti* were determined by using the bioassay system described by Pridgeon et al. [68]. Eggs were hatched and neonates were held overnight in the laboratory. To conduct the bioassay, 5 1-d-old larvae were transferred into each well of 24-well plates (BD Labware, Franklin Lakes, NJ, USA) using a Pasteur pipette. Larvae were fed on a diet (2% slurry of 3:2 Beef Liver powder and Brewer’s yeast), l at a rate of 50 µL/well. Treatments were prepared in dimethyl sulfoxide (DMSO) which was used as a solvent control. Applications were applied in a volume of 11 µL per well whereas DMSO was applied as a control. To ensure the proper mixing of the chemicals, the plates were swirled clockwise and counterclockwise, front and back, and side to side five times. Mortality in each treatment was recorded at 24 h post treatment. Larvae that did not show any movement after manual disturbance of water were noted as dead. Five concentrations ranging between 125 to 7.8 ppm for the essential oils and 100 to 12.5 ppm for pure compounds were tested. Permethrin was the positive control and the treatments were replicated 10 times.

#### 5.2.4. Statistical Analyses

In order to make direct comparisons among test samples and to compensate for variation in overall response among replicates, biting deterrent activity was calculated as the Biting Deterrence Index (BDI) [46]. The BDIs were calculated using the following formula:

$$\left[{\mathrm{BDI}}_{i,j,k}\right]=\left[\frac{\begin{array}{ccc} {\mathrm{PNB}}_{i,j,k} & & {\mathrm{PNB}}_{c,j,k} \end{array}}{\begin{array}{ccc} {\mathrm{PNB}}_{d,j,k} & - & {\mathrm{PNB}}_{c,j,k} \end{array}}\right]$$

where PNB*_i_*_,*j*,*k*_ denotes the proportion of females not biting when exposed to test compound *i* for replication *j* and day *k* (*i* = 1–4, *j* = 1–5, *k* = 1–2), PNB*_c_*_,*j*,*k*_ denotes the proportion of females not biting in solvent control “*c*” for replication *j* and day *k* (*j* = 1–5, *k* = 1–2) and PNB*_d_*_,*j*,*k*_ denotes the proportion of females not biting in response to DEET “*d*” (positive control) for replication *j* and day *k* (*j* = 1–5, *k* = 1–2). This formula accounts for inter-day variation and incorporates information from both solvent and positive control.

BDI values not significantly different from 1 are similar to DEET. Data were analyzed using SAS Proc ANOVA (SAS Institute 2012). To determine whether confidence intervals include the values of 0 or 1 for treatments, Scheffe’s multiple comparison procedure with the option of CLM was used. LC_50_ values for larvicidal data were calculated by using Proc Probit (SAS 2012 Cary, NC, USA).

### 5.3. Imported Fire Ants

RIFA, BIFA and HIFA workers were used in this study. HIFA was used from natural mounds, located at the University Field Station, University of Mississippi, 15 County Road 2078, Abbeville, MS 38601, USA whereas RIFA and BIFA colonies were brought from Washington County, MS 38748, USA (33°09′31.2″ N 90°54′56.4″ W) and Tunica County, MS-713, Hernando, MS 38632, USA (34°49′56.5″ N 90°12′55.6″ W), respectively. The ants were maintained at the laboratory under settings of 28 ± 2 °C temperature, and 50 ± 10% relative humidity. The ants were kept inside plastic trays and fed with crickets and a 25% sugar water solution. Moist sand was the digging substrate and a partially water-filled test tube plugged with cotton was used as a water source. The sides of the trays containing fire ants were coated with Insect a Slip (BioQuip Products 2321 Gladwick Street Rancho Dominguez, CA 90220, USA) to prevent the ants from escaping. Fire ants were kept in the laboratory for one month before starting the bioassays. Venom alkaloid and cuticular hydrocarbon profiles were used to identify the fire ant species and hybrids, following Ross et al. [69].

#### 5.3.1. Digging Bioassay

Repellency of *B. microdonta* essential oil against RIFA, BIFA and HIFA workers was determined using a bioassay described by Ali et al. [17]. In the construction and maintenance of mounds, fire ant digging activity is very crucial. Fire ants as per their normal behavior dig through sand while repellents prevent the digging in treated materials. This bioassay uses the digging behavior of ants as a basis to determine the repellency. In this bioassay, arena Petri dishes were used whose inner walls were treated with Insect a Slip. As a digging substrate, de-ionized water-washed dried sand of uniform 500-micron size was used. A 4 g of sand in a 45 mL size fluted aluminum weighing dish (Fisher Scientific, 300 Industry Drive Pittsburgh, PA 15275, USA) was weighed and treated in a volume of 400 µL and thoroughly mixed well with a small spatula. The solvent ethanol was evaporated at room temperature. A 0.65 µL/g of de-ionized water was added to sand in each treatment to moisten it. The vials were filled with treated sand and screwed to vial caps at the bottom of the arena. Each vial contained a mean weight of 3.6 g of dry sand. Using a camel hairbrush and feather forceps, about fifty ant workers were gently released in the middle portion of the arena petri dish. Laboratory conditions were set at 25 ± 2 °C temperature and 50 ± 10% RH. At 24 h post treatment, sand was collected back into aluminum dishes and weighed after oven drying at 150 °C for 1 h. The highest screening dose tested was 125 ppm, which was diluted serially until the data showed repellency similar to solvent control. Experiments were run in triplicates on different days using a separate batch of ants. To compute significant effects, the data were subjected to Analysis of Variance (ANOVA), followed by Ryan–Einot–Gabriel–Welsch multiple range test at a level of *p* ≤ 0.05 (SAS 9.4 2012).

#### 5.3.2. Toxicity Bioassay

Toxicity of *B. microdonta* against the workers of RIFA, BIFA and HIFA was determined by using a toxicity bioassay described by Ali et al. [17]. A 3 g of sand was treated in a volume of 300 µL in aluminum fluted 42 mL dishes. On evaporation of ethanol, we added 0.65 µL/g of de-ionized water to the sand to moisten it and transferred it into a 60 × 15 mm petri dish lined with Insect a Slip. Using a camel hairbrush and forceps, ten worker ants were released per Petri dish. A water-soaked cotton swab tip was placed in Petri dishes one hour after releasing the ants to avoid dehydration. The dead ants were counted at 24 h post treatment. Dosages ranging between 625 and 39 µg/g were used in this study. LC_50_ values were calculated by subjecting mortality data to Probit analysis (SAS 2012).

## Figures and Tables

**Table 1:** Toxicity of the essential oils of *Baccharis* species and its major constituents against 1 d old larvae of *Aedes aegypti* at 24 h post-treatment.

EO/Compound	*n* *	Slope ± SE	LC_50_ ppm (95% CI) **	LC_90_ ppm (95% CI)	χ^2^	*df*
*B. reticularioides*	50	2.3 ± 0.32	84.4 (75.1–95.5)	147.3 (124.8–191.9	50.5	48
*B. pauciflosculosa*	50	1.9 ± 0.21	31.9 (28.1–36.3)	63.2 (53.3–80.1)	82.1	48
*B. sphenophylla*	50	2.2 ± 0.25	30.8 (27.4–34.8)	55.5 (47.3–70.0)	7.6	48
*B. punctulata*	50	2.0 ± 0.23	20.4 (18.0–23.1)	38.8 (33.0–49.0)	75.6	48
*B. microdonta*	50	1.35 ± 0.14	28.6 (24.5–33.3)	73.4 (59.4–98.4)	95.0	48
Kongol	50	1.94 ± 0.22	32.3 (30.3–39.1)	62.1 (52.5–78.9)	78.1	38
Spathulenol	50	1.99 ± 0.24	48.7 (43.1–55.6)	92.7 (77.6–120.8)	67.2	38

* *n* is the number of larvae treated. ** CI—confidence interval. *df*—degrees of freedom.

**Table 2:** Mean weight (g) of treated sand removed by the red imported fire ant workers released in multiple-choice digging bioassay with different concentrations of *Baccharis microdonta* essential oil and DEET.

Conc. (µg/g)	Sand Removed ± SE *	*F*-Value	*p*-Value	Conc. (µg/g)	Sand Removed ± SE	*F*-Value	*p*-Value	Conc. (µg/g)	Sand Removed ± SE	*F*-Value	*p*-Value
**RIFA**				**BIFA**				**HIFA**			
**Essential oil**											
Control	1.05 ± 0.20 A	3.17	0.085	Control	1.30 ± 0.16 A	3.14	0.0868	Control	2.25 ± 0.41 A	3.77	0.0593
2.4	0.50 ± 0.16 A			2.4	0.41 ± 0.24 A			19.5	0.59 ± 0.17 A		
1.2	0.48 ± 0.02 A			1.2	0.91 ± 0.27 A			9.8	0.56 ± 0.08 A		
0.6	0.71 ± 0.15 A			0.6	0.93 ± 0.16 A			4.8	1.59 ± 0.12 A		
Control	1.80 ± 0.18 A	15.39	0.001	Control	1.20 ± 0.23 A	15.39	0.0011	Control	2.34 ± 0.22 A	22.94	0.0003
19.5	0.61 ± 0.03 B			19.5	0.003 ± 0.003 B			156	0.14 ± 0.11 B		
9.8	1.00 ± 0.13 B			9.8	0.18 ± 0.16 B			78	0.32 ± 0.18 B		
4.9	0.64 ± 0.17 B			4.9	0.33 ± 0.31 B			39	0.66 ± 0.29 B		
**DEET**											
Control	1.43 ± 0.19 A	14.86	0.001	Control	2.37 ± 0.30 A	34.85	0.0001	Control	1.45 ± 0.19 A	19.83	0.0005
156	0.08 ± 0.04 C			156	0.32 ± 0.27 C			156	0.04 ± 0.43 B		
78	0.74 ± 0.18 B			78	1.43 ± 0.12 B			78	0.24 ± 0.20 B		
39	1.13 ± 0.10 AB			39	2.06 ± 0.08 A			39	1.11 ± 0.12 A		

* Sand removed is in grams. Means within a column, in an experiment, sharing the same letter are not significantly different (Ryan–Einot–Gabriel–Welsch multiple range test; *p* > 0.05).

**Table 3:** Toxicity of *Baccharis microdonta* essential oil against RIFA, BIFA and HFA workers at 24 h post treatment.

E. Oil/Compound	*n* *	Slope ± SE	LC_50_ (95% CI) **	LC_90_ (95% CI)	χ^2^	*df*
RIFA						
*B. microdonta*	30	3.08 ± 0.58	78.9 (68.7–90.2)	119.7 (102.5–158.3)	28.3	13
Bifenthrin	40	1.21 ± 0.18	0.03 (0.023 ± 0.04)	0.09 (0.06 ± 0.16)	42	19
BIFA						
*B. microdonta*	30	2.58 ± 0.56	97.1 (76.8–126.7)	159.5 (123.2–282.9)	21	13
Bifenthrin	40	1.36 ± 0.23	0.032 (0.023 ± 0.044)	0.08 (0.06 ± 0.15)	34	19
HIFA						
*B. microdonta*	30	1.93 ± 0.36	136.5 (108.2–174.3)	265.2 (201.4–453.8)	29.4	13
Bifenthrin	40	0.86 ± 0.13	0.018 (0.013 ± 0.024)	0.07861 (0.05 ± 0.17)	42.4	22

* *n* denotes the number of workers used in each treatment. ** LC_50_ and LC_90_ values are in µg/g and Cis are confidence intervals. *df*—degrees of freedom.

## Data Availability

All data from this study are included within this manuscript.

## Supplementary Materials

The following supporting information can be downloaded at: https://www.mdpi.com/article/10.3390/jox13040041/s1, Table S1: chemical compositions of the essential oils of Baccharis species. References [70, 71] are cited in Supplementary Materials.
