# Supplementary material for: Baccharis Species Essential Oils: Repellency and Toxicity against Yellow Fever Mosquitoes and Imported Fire Ants
Source: J Xenobiot. 2023 Oct 27;13(4):641–52. doi: 10.3390/jox13040041 (PMC10660731; doi:10.3390/jox13040041)
Supplement: Supplementary file 1 [file jox-13-00041-s001.zip › jox-2627185-supplementary.pdf]

S1 Table. Chemical compositions of the essential oils of *Baccharis* species.

No.	RRI[a]	Compound name	Peak Area %[b]					ID
			B. microdonta	*B. pauciflosculosa*	*B. punctulata*	*B. reticularioides*	*B. sphenophylla*	
1	922	β-Thujene	-	3.420	0.412	0.889	0.534	MS
2	928	**α-Pinene**	0.753	10.437	3.548	**24.477**	**10.709**	t_R, MS
3	943	Camphene	-	0.776	0.186	0.431	0.384	t_R, MS
4	947	Thuja-2,4(10)-diene	-	-	-	2.911	0.120	MS
5	967	Sabinene	-	2.745	0.892	0.392	3.809	t_R, MS
6	971	**β-Pinene**	2.193	**18.317**	4.947	7.671	**15.197**	t_R, MS
7	985	Carveol	-	-	-	0.669	-	t_R, MS
8	987	β-Myrcene	-	3.642	0.297	0.273	0.663	t_R, MS
9	1001	p-Mentha-1,3,8-triene	-	-	-	0.573	-	MS
10	1002	α-Phellandrene	-	-	0.325	-	-	t_R, MS
11	1004	3-Carene	-	-	-	-	0.986	t_R, MS
12	1013	α-Terpinene	-	0.190	0.104	0.428	0.580	t_R, MS
13	1020	p-Cymene	-	0.818	3.437	3.175	1.309	t_R, MS
14	1024	**Limonene**	1.052	**18.751**	11.350	2.472	**14.488**	t_R, MS
15	1043	Ocimene	-	-	0.251	0.140	-	MS
16	1053	γ-Terpinene	-	0.314	0.162	0.698	1.030	t_R, MS
17	1080	Terpinolen	-	0.136	-	0.439	0.371	t_R, MS
18	1085	p-Cymenene	-	-	-	0.848	0.108	MS
19	1098	Linalool	0.141	0.413	0.309	-	0.151	t_R, MS
20	1112	Thujone	-	-	-	0.596	-	t_R, MS
21	1121	α-Campholenal	-	-	-	1.627	-	MS
22	1131	Nopinone	0.121	-	-	0.377	-	MS
23	1133	*trans*-Pinocarveol	0.765	0.413	0.266	4.437	0.788	t_R, MS
24	1140	*trans*-Verbenol	-	0.103	0.110	0.943	0.112	MS
25	1145	Unknown	-	-	-	2.116	-	MS
26	1153	2-Norpinanone, 3,6,6-trimethyl-	-	-	-	0.520	-	MS
27	1155	Pinocarvone	0.341	0.260	0.160	0.802	0.268	t_R, MS
28	1167	α-Phellandren-8-ol	0.148	-	-	5.597	0.149	MS
29	1174	Terpinen-4-ol	-	1.187	0.466	1.340	3.096	t_R, MS
30	1182	p-Cymen-8-ol	-	0.114	-	0.833	0.207	MS
31	1187	Myrtenal	0.620	0.340	0.240	2.135	0.525	t_R, MS
32	1190	α-Terpineol	1.008	0.920	0.420	4.817	1.843	t_R, MS
33	1200	Verbenone	-	-	-	2.941	-	t_R, MS
34	1214	*cis*-Carveol	0.151	0.149	0.274	1.186	0.269	t_R, MS
35	1238	Carvone	0.181	0.130	0.415	0.430	0.215	t_R, MS
36	1242	2-Caren-4-ol	-	-	-	0.321	-	MS
37	1279	Bornyl acetate	-	-	1.317	0.596	0.118	t_R, MS
38	1366	α-Copaene	0.272	0.222	-	-	0.258	MS
39	1379	β-Cubebene		0.335	-	-	0.133	MS
40	1381	β-Elemen	0.932	0.412	0.666	-	0.234	MS
41	1401	β-Caryophyllen	1.029	1.797	3.346	-	3.598	t_R, MS
42	1409	α-Humulene	0.445	0.189	0.419	-	0.311	t_R, MS
43	1410	Alloaromadendrene	0.170	0.525	0.154	0.176	0.266	MS
44	1413	δ-Cadinene	0.362	0.123	-	-	0.103	MS
45	1414	γ-Muurolene	0.151	0.262	0.168	-	0.268	MS
46	1414	α-Gurjunene			0.422	-	-	MS
47	1415	β-Copaene	0.611	2.554	3.626	-	1.441	MS
48	1416	β-Guaiene	1.430	0.154	0.120	0.152	0.242	MS
49	1417	Ledene	0.227	0.293	0.130	-	0.174	t_R, MS

50	1418	Bicyclogermacrene	-	1.249	3.099	-	0.475	MS
51	1418	α-Selinene	0.620	-	-	-	-	MS
52	1419	α-Muurolene	0.125	0.626	0.262	-	0.285	MS
53	1422	β-Bisabolene	-	-	1.183	-	-	MS
54	1422	γ-Cadinene	0.116	0.391	-	0.561	0.513	MS
55	1423	β-Cadinene	0.964	2.739	1.133	0.276	0.785	MS
56	1424	*cis*-Calamenene	0.166	0.294	-	-	-	MS
57	1428	α-Calacorene	0.790	-	-	0.402	0.184	MS
58	1430	Unknown	0.265	-	0.224	-	0.207	MS
59	1431	2-Butenal, 2-methyl-4-(2,6,6-trimethyl-1-cyclohexen-1-yl)-	0.379	-	-	-	-	MS
60	1431	Elemol	-	0.168	1.018	-	-	MS
61	1433	(1R,7S,E)-7-Isopropyl-4,10-dimethylenecyclodec-5-enol	0.832	-	-	0.333	0.343	MS
62	1436	Palustrol	3.336	-	0.126	-	-	MS
63	1438	**Spathulenol**	**23.274**	9.520	9.962	5.519	**13.112**	t_R, MS
64	1439	Caryophyllene oxide	5.401	2.111	5.301	1.373	5.323	t_R, MS
65	1440	Unknown	0.591	-	-	-	0.354	MS
66	1442	Aristolene epoxide	0.144	0.201	0.142	0.127	0.257	MS
67	1443	Viridiflorol	3.850	1.804	0.174	-	1.654	t_R, MS
68	1446	Ledol	2.819	-	-	-	0.102	MS
69	1448	Calarene epoxide	1.545	0.166	0.428	0.512	0.409	MS
70	1451	Unknown	1.491	-	-	-	0.366	MS
71	1455	Cubenol	1.027	0.375	-	0.142	0.170	MS
72	1456	Isospathulenol	-	-	1.087	-	-	MS
73	1457	γ-Himachalene	-	0.147	0.380	-	0.237	MS
74	1457	Unknown	1.370	-	-	-	-	MS
75	1458	δ-Cadinol	0.445	-	1.946	0.109	0.145	MS
76	1460	τ-Cadinol	0.419	0.767	0.261	2.640	2.264	MS
77	1461	*epi*-α-Muurolol	0.178	0.651	0.272	-	-	MS
78	1462	Alloaromadendrene oxide	0.874	-	-	-	-	MS
79	1464	β-Eudesmol	-	-	0.640	-	-	t_R, MS
80	1464	α-Bisabolol oxide B	-	-	1.168	-	-	MS
81	1464	α-Cadinol	-	1.438	-	1.361	1.481	MS
82	1465	**Kongol**	**22.434**	-	-	-	-	t_R, MS
83	1467	4H-Naphth[1,2-b]oxireno[c]furan, 2,2a,5,5a,6,7,8,9b-octahydro-2a,5a,9-trimethyl-, (2aS,3aR,5aS,9bR)-	-	-	1.338	-	-	MS
84	1467	Murolan-3,9(11)-diene-10-peroxy	0.400	-	-	-	-	MS
85	1469	Isoaromadendrene epoxide	3.046	0.207	0.488	0.461	0.766	MS
86	1473	Unknown	0.579	-	-	-	-	MS

87	1474	(1R,7S,E)-7-Isopropyl-4,10-dimethylenecyclodec-5-enol	0.770	0.389	-	0.146	0.424	MS
88	1475	α-Bisabolol	-	-	**23.633**	-	-	t_R, MS
89	1480	6-Isopropenyl-4,8a-dimethyl-1,2,3,5,6,7,8,8a-octahydro-naphthalen-2-ol	0.688	-	-	-	-	MS
90	1483	Unknown	0.664	-	-	-	-	MS
91	1484	Ylangenal	0.764	-	-	-	-	MS
92	1485	1,3-di-iso-propylnaphthalene	-	-	0.434	-	-	MS
93	1489	Aromadendrene oxide	0.306	-	-	-	-	MS
94	1493	Murolan-3,9(11)-diene-10-peroxy	0.356	-	0.103	-	-	MS
Compounds identified (%)			94.131	93.684	93.741	92.39	94.913	
Monoterpenoids hydrocarbons			7.530	22.484	22.567	28.283	25.423	
Oxygenated monoterpenoids			15.061	16.863	15.624	33.939	18.644	
Sesquiterpenoids hydrocarbons			30.122	31.852	22.567	9.427	27.118	
Oxygenated sesquiterpenoids			41.418	22.484	31.247	20.741	23.728	
Others			-	-	1.736	-	-	

RRI: relative retention indices; RRI a, relative retention indices calculated against n-alkanes on the DB-5MS column; RI lit b, retention index literature (DB-5 column) [70]; RRI c, relative retention indices calculated against n-alkanes on the DB-WAX column; RI lit d, retention index literature (CW20M column) [71], Peak Area% e; stereoisomers not identified f; NC, non-polar column; PC, polar column, tR, identification based on the retention times (tR) of genuine compounds on the DB-5MS column; MS, identified on the basis of computer matching of the mass spectra with those of the Wiley and NIST libraries and comparison with literature data. The compounds in bold represent the major compounds.

Note: This table was previously published as part of the paper: Budel JM, Wang M, Raman V, Zhao J, Khan SI, Rehman JU, Techen N, Tekwani B, Monteiro LM, Heiden G, Takeda IJM, Farago PV, Khan IA. Essential oils of five *Baccharis* species: investigations on the chemical composition and biological activities. Molecules. 2018 Oct 12;23(10):2620. doi: 10.3390/molecules23102620.

Supplementary references:

70. Adams, R. P., *Identification of Essential Oil Components by Gas Chromatography/Mass Spectrometry*, 4th ed.; Allured Publishing Corporation: Carol Stream, IL, USA, 2007; ISBN 978-1-932633-21-4. Supplementary reference

71. Davies, N., Gas chromatographic retention indices of monoterpenes and sesquiterpenes on methyl silicon and Carbowax 20M phases. J. Chromatogr. A. **1990**, 503, 1–24. doi: 10.1016/S0021-9673(01)81487-4. Supplementary reference
